# Supplementary material for: Probing the Structural Determinants of Amino Acid Recognition: X-Ray Studies of Crystalline Ditopic Host-Guest Complexes of the Positively Charged Amino Acids, Arg, Lys, and His with a Cavitand Molecule
Source: Molecules. 2018 Dec 19;23(12):3368. doi: 10.3390/molecules23123368 (PMC6321202; doi:10.3390/molecules23123368)
Supplement: Supplementary file 1 [file molecules-23-03368-s001.pdf]

molecules

SUPPLEMENTARY MATERIAL

Probing the structural determinants of amino acid recognition: X-ray studies of crystalline ditopic host-guest complexes of the positively charged amino acids, Arg, Lys and His with a cavitand molecule.

Giovanna Brancatelli[1], Enrico Dalcanale[2], Roberta Pinalli[2,*] and Silvano Geremia[1,*]

Table S1. Experimental details for the co-crystallization experiments of the host-guest complexes of cavitand Tiiii[H, CH_3, CH_3] with the selected amino acids.

**Amino acid**	**Amino acid Concentration in HCl 1 M(mM)**	**Molar Ratio cavitand:amino acid**	**Composition of Reservoir (v/v)**
Arg	300	1:5	PEG 300 25-50%
Lys	30	2:1	PEG 300 5-15%
His	300	1:5	PEG 300 25-50%

Table S2. Crystal data and structure refinement details for the host-guest complexes

	**Arg 2HCl**	**Lys 2HCl**	**His 2HCl**
Empirical formula	2($C_{36}H_{36}O_{12}P_4$), $C_6H_{16}N_4O_2$, 2Cl, 4.4($C_2H_3F_3O$), 3.7H_2O	2($C_{36}H_{36}O_{12}P_4$), $C_6H_{16}N_2O_2$, 2Cl, 6.1($C_2H_3F_3O$), 2.8H_2O	4($C_{36}H_{36}O_{12}P_4$), 2($C_6H_{11}N_3O_2$), 4Cl 10($C_2H_3F_3O$), 4 H_2O
Formula weight	2253.06	2158.80	4546.81
T (K)	100(2)	100(2)	100(2)
λ (Å)	0.700	0.700	0.700
Crystal system	Monoclinic	Orthorhombic	Monoclinic
Space group	$P\,2_1$	$P\,2_12_12$	$P\,2_1$
Unit cell dimensions (Å, °)	a = 12.443(1)<br>b = 26.937(2)<br>c = 17.394(2)<br>β = 108.96(3)	a = 21.654(4)<br>b = 32.439(7)<br>c = 15.809(3)	a = 17.644(2)<br>b = 23.627(2)<br>c = 25.567(3)<br>β = 93.909(4)
V (Å3)	5513.8(13)	11105(4)	10633(2)
Z	2	4	2
ϱ(calc) (g/mm^3)	1.399	1.464	1.469
μ (mm^{-1})	0.212	0.207	0.263
F(000)	2406	5060	4816
Reflections collected	18443	44450	28901
Independent reflections	18443	17357	28901
Data / restraints / parameters	18443/3/1288	17357/18/1128	28901/1753/2674
GooF	1.066	1.008	1.006
R_1, wR_2 [I>2σ(I)]	0.0749, 0.2095	0.0684, 0.1882	0.0682, 0.1548
R_1, wR_2 (all data)	0.0763, 0.2113	0.1014, 0.2112	0.1219, 0.1868
Absolute structure parameter	0.10(2)	0.09(6)	-0.03(8)
CCDC code number	1869187	1876524	1869186
